# Clinical characterization of membrane oxygenator exhaust capnometry during venovenous extracorporeal membrane oxygenation

**DOI:** 10.1038/s41598-026-51104-x

**Published:** 2026-05-07

**Authors:** John W. Stokes, Whitney D. Gannon, Elizabeth Simonds, Ioannis A. Ziogas, W. Kelly Wu, Yatrik J. Patel, Sean A. Francois, Caitlin T. Demarest, Matthew Bacchetta, Rei Ukita

**Affiliations:** 1 Department of Thoracic Surgery, Vanderbilt University Medical Center, Nashville, TN USA; 2 Department of Pulmonary, Allergy, and Critical Care Medicine, Vanderbilt University Medical Center, Nashville, TN USA; 3 Vanderbilt University School of Medicine, Nashville, TN USA; 4 Department of Surgery, University of Colorado Anschutz Medical Campus, Aurora, USA; 5 Transplant Center, Cleveland Clinic, Cleveland, OH USA; 6 Department of Cardiac Surgery, Vanderbilt University Medical Center, Nashville, TN USA; 7 Department of Surgery, Maine Medical Center, Portland, ME USA; 8 Department of Biomedical Engineering, Vanderbilt University, Nashville, TN USA

**Keywords:** Diseases, Health care, Medical research, Physiology

## Abstract

Capnometry is a cost-efficient method of monitoring carbon dioxide (CO_2_) in healthcare settings, especially during mechanical ventilation and cardiopulmonary bypass. However, the utility of capnometers for studying CO_2_ elimination in a longer-duration, clinical extracorporeal membrane oxygenation (ECMO) has been poorly characterized. This report presents data from 10 venovenous ECMO patients at Vanderbilt University Medical Center between April and June 2021. Exhaust CO_2_ was intermittently measured from a membrane oxygenator’s gas outlet, and arterial blood samples were simultaneously drawn from the patient. Clinical variables including ECMO and ventilator management, patient demographics, and outcomes were recorded. Exhaust CO_2_ and other study variables were recorded at 233 discrete time points from the 10 patients. The median duration of ECMO support was 9 days. Sweep gas flow rate demonstrated a strong, negative correlation with exhaust CO_2_ concentration, from both univariate and multivariate regression analyses (adjusted β = − 5.556 for sweep < 4 L/min, *p* < 0.001). Blood flow, oxygenator duration of use, and arterial CO_2_ demonstrated weaker relationships with exhaust CO_2_ (adjusted β = 2.341, − 0.258, 0.175, respectively), which agrees with the clinical understanding about CO_2_ management during ECMO. This descriptive study provides foundation for future research to precisely define the clinical role of ECMO exhaust capnometry in the management of the sweep gas flow and the overall therapy.

## Introduction

Capnometry is a cost-efficient technology for non-invasively monitoring the concentration of CO_2_ in gas. In health care settings, it has been used to measure CO_2_ concentration in exhaled gas during invasive and non-invasive ventilation applications^1,2^, confirmation of advanced airway placement, and during cardiopulmonary resuscitation^3,4^. Analogous to exhaled gas from the native lung, the gas flow leaving the gas outlet of the membrane oxygenator (exhaust gas) in extracorporeal life support (ECLS) circuits has a measurable concentration of CO_2_^5,6^. Several applications for exhaust capnometry during ECLS have been proposed. Exhaust CO_2_ measurements inform membrane oxygenation CO_2_ transfer^7,8^ thereby providing insight into the health and efficiency of oxygenators^8,9^. Changes in exhaust CO_2_ concentration may identify circuit thrombosis^10^, disruptions in sweep gas flow, patient factors, and condensation accumulation within the gas exchange chamber of membrane oxygenators^7^. Exhaust CO_2_ measurements may inform sweep gas flow titration^11,12^ and have been applied for autoregulatory control of sweep gas flow in large animals^13^. Newer ECLS devices are incorporating means of monitoring membrane oxygenator exhaust CO_2_^11,14^.

Despite the potential uses for this non-invasive measurement, the relationship between exhaust CO_2_ and the important variables that may inform its clinical application are inadequately understood. Changes in exhaust CO_2_ concentration over the duration of membrane oxygenator use and the effect of the partial pressure of CO_2_ in patients’ blood have not been described in human subjects receiving extracorporeal membrane oxygenation (ECMO). Exhaust capnometry is rarely monitored during ECMO or incorporated into clinical decision-making^15^, and data informing ECMO applications are not available. To identify the role of exhaust CO_2_ monitoring in the care of a patient receiving ECMO, these relationships must first be explored.

**Fig. 1:**
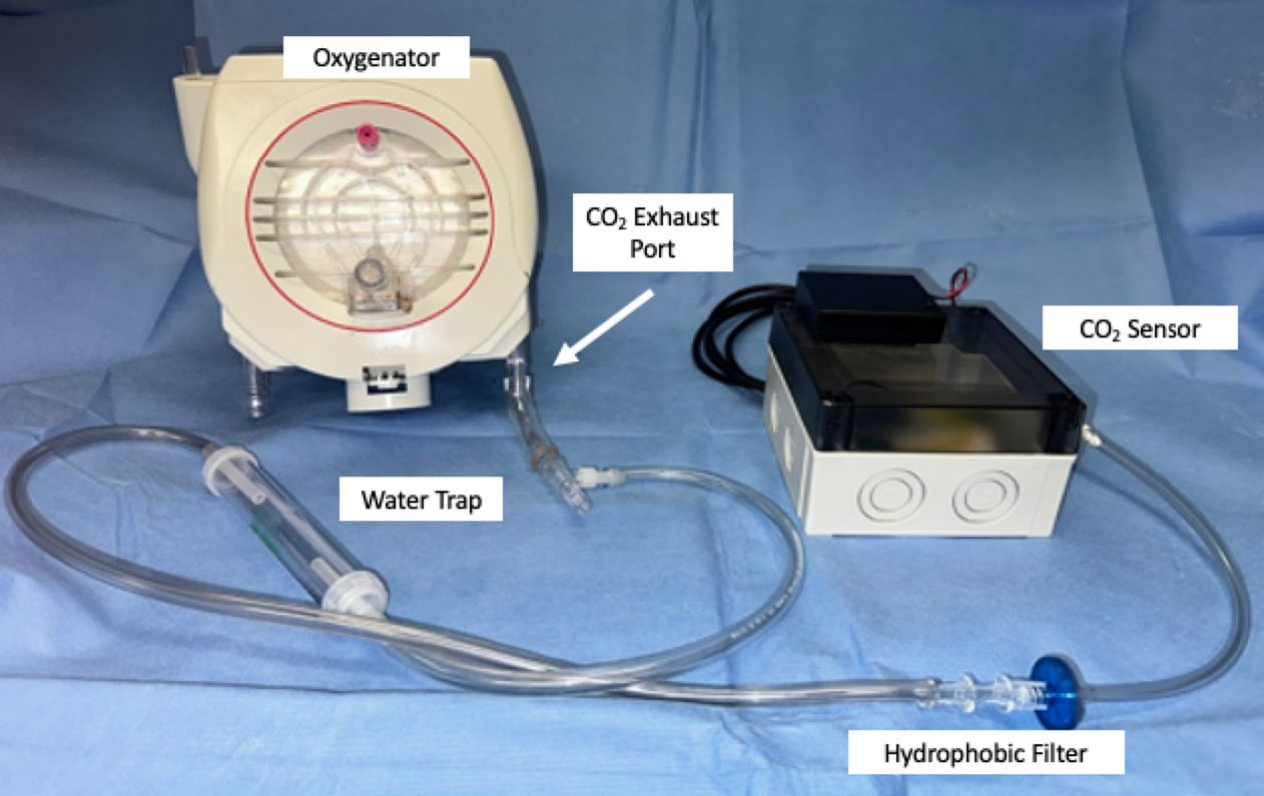
Exhaust CO_2_ measurement equipment and integration with ECMO circuits. Exhaust gas was sampled in a side-stream fashion via a stop-cock connecter and ¼-inch tubing. A water trap and hydrophobic filter were integrated into the sampling tubing to avoid water contamination of the CO_2_ gas analyzer device.

This study measured exhaust CO_2_ concentration from membrane oxygenators (Fig. 1) and concomitantly recorded clinically relevant variables among patients receiving venovenous ECMO (VV-ECMO). The purpose is to examine the relationship between CO_2_ removal, and ECMO circuit and patient variables and assess the potential utility of oxygenator capnometry for longitudinal patient and/or device monitoring. We hypothesized that CO_2_ removal would decrease over the duration of oxygenator use and increase with higher arterial partial pressure of CO_2_, holding other relevant variables constant. Based on prior work, we also expected a negative and non-linear correlation between sweep gas flow rate and exhaust CO_2_ concentration^11,16,17^.

## Results

### Participant and device characteristics

Ten VV-ECMO patients were enrolled in this study. The median age of study participants was 36 years old (IQR 29-47), as shown in Table 1. Nine patients (90%) received ECMO for acute respiratory distress syndrome (ARDS) secondary to COVID-19. All patients were receiving invasive mechanical ventilation at every exhaust CO_2_ measurement time point, and the median duration of mechanical ventilation of participants was 27 days (IQR 12–70 days). Femoral-internal jugular dual-site configuration was the initial configuration in all 10 patients; one patient was reconfigured to an internal jugular vein single-site configuration during study participation. Among the 10 patients included in the study, 16 distinct oxygenators were used since 2 of the patients required repeated device exchange. Out of the 16 oxygenators studied, 8 were (50%) Cardiohelp devices, and 8 (50%) were Nautilus oxygenators coupled with CentriMag blood pumps. Median total oxygenator duration was 10 days (IQR 7–12). Median ICU and hospital length of stay were 29 days (IQR 11–74 days) and 41 days (IQR 21–78 days), respectively. A total of 6 patients (60%) survived to both decannulation and hospitalization.

**Table 1:** Characteristics of patients who enrolled in the study and their devices.

	All participants (N = 10)
Patient characteristics	
Age (years)	36 (29–47)
Female	5 (50)
Body mass index (kg^2^)	41.8 (35.7–49.2)
Body surface area (m^2^)	2.4 (2.2–2.6)
ECMO indication	
Acute respiratory distress syndrome	10 (100)
COVID-19	9 (90)
Aspiration pneumonitis	1 (10)
Initial dual-site V-V ECMO	10 (100)
Duration of mechanical ventilation (days)	27 (12–70)
Duration of ECMO (days)	9 (5–63)
Study oxygenators per patient	1 (1–1)
Measurement per patient	11 (8–41)
ICU length of stay (days)	29 (11–74)
Hospital length of stay (days)	41 (21–78)
Survival to ECMO decannulation	6 (60)
Hospital survival	6 (60)

	All Oxygenators (N = 16)
Oxygenator characteristics	
Measurement per oxygenator	11 (8–15)
Oxygenator duration (days)	10 (7–12)
Oxygenator type	
Cardiohelp	8 (50.0)
Cardiohelp duration (days)	8 (6–13)
Study time point measurements	108
Nautilus	8 (50.0)
Study time point measurements	125
Nautilus duration	11 (9–12)

Data are presented as frequency with percentage or median with interquartile range

### Longitudinal measurements of exhaust CO_2_ and concomitantly recorded variables

**Fig. 2:**
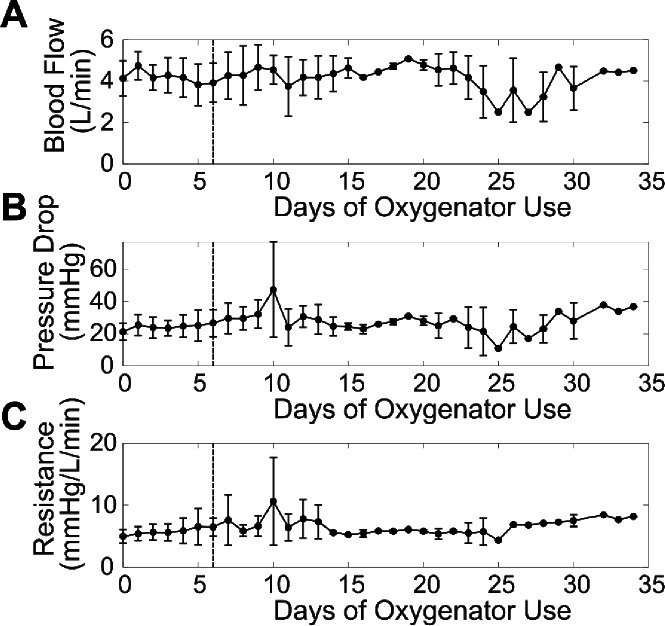
Longitudinal measurements of blood flow, oxygenator pressure drop, and resistance over the course of oxygenator use. A vertical dashed line represents the 6-day mark used for stratification in subsequent statistical analyses.

**Fig. 3:**
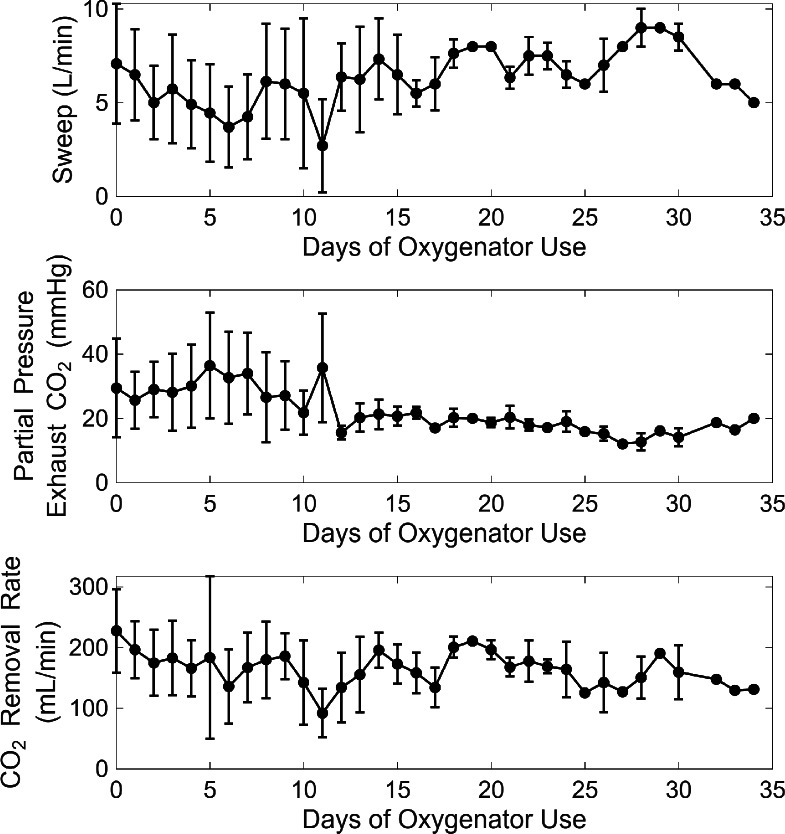
Longitudinal measurements of sweep gas flow rate, exhaust CO_2_, and CO2 removal rate over the course of oxygenator use. A vertical dashed line represents the 6-day mark used for stratification in subsequent statistical analyses.

**Fig. 4:**
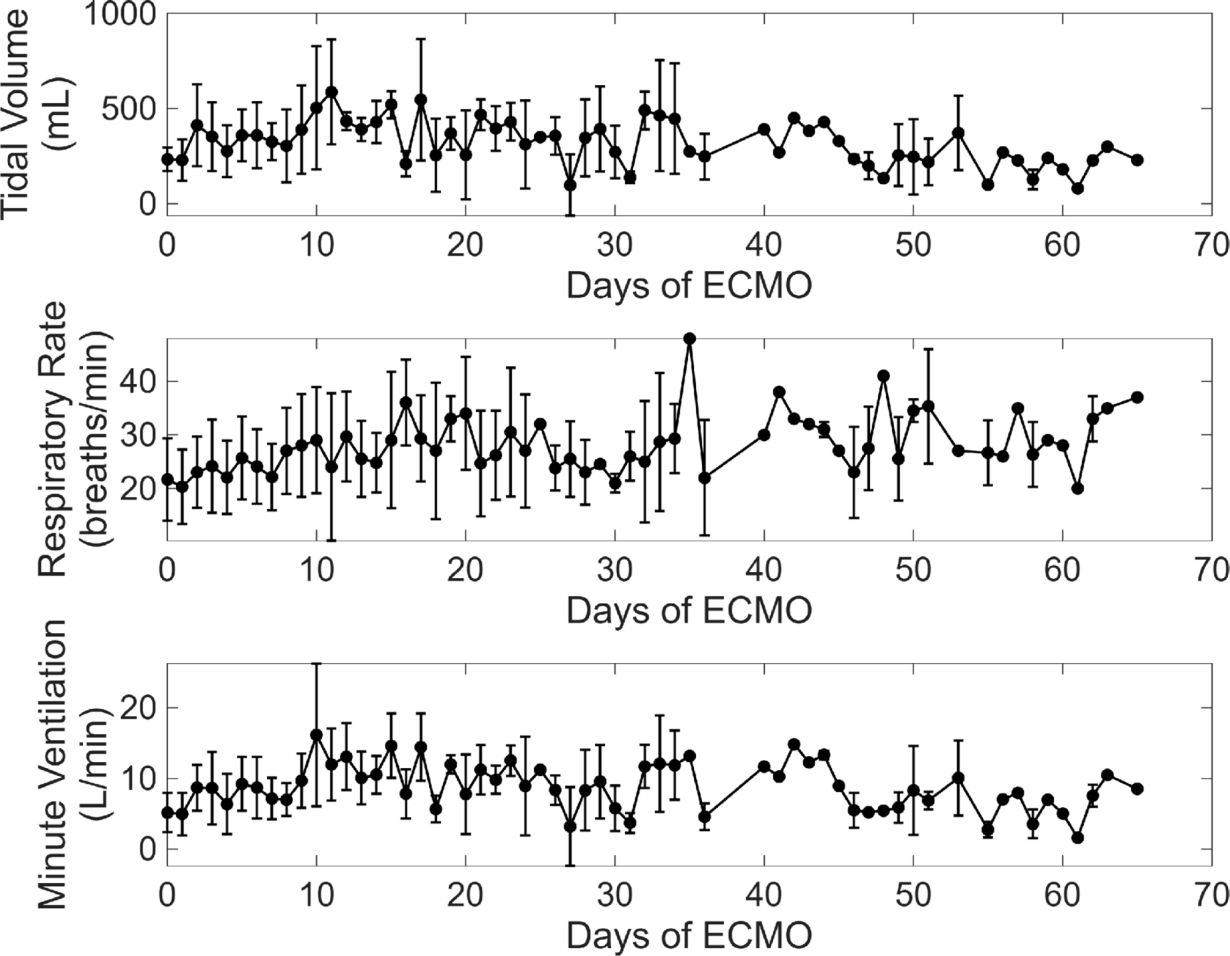
Longitudinal measurements of tidal volume, respiratory rate, and minute ventilation over the course of ECMO therapy.

Exhaust CO_2_ and other study variables were recorded at 233 discrete time points across 10 patients. Figure 2 shows the trajectories of ECMO blood flow, oxygenator pressure drop, and resistance over the days of oxygenator use, and Fig. 3 shows the sweep gas, exhaust CO_2_, and CO_2_ removal rate over the same course. The median exhaust CO_2_ concentration was 22.4 mmHg (IQR 18.1–33.0 mmHg). Median ECMO blood flow rate and sweep gas flow rate were 4.40 LPM (IQR 3.53–5.05 LPM) and 5.0 LPM (IQR 4.0–8.0 LPM), respectively. A total of 196 measurements (84.1%) were obtained while the fraction of delivered oxygen in the sweep gas flow was 1.0. Median oxygenator duration at the time of exhaust CO_2_ measurement was 6.02 days (IQR 2.81–10.34 days). The median partial pressure of arterial CO_2_ at the time of exhaust CO_2_ measurement was 50 mmHg (IQR 45–61 mmHg). Data stratified by the duration of oxygenator use longer or shorter than 6 days are presented in Table 2. Between the two strata, there were no significant differences in blood flow rate, sweep gas flow rate, or the fraction of delivered oxygen. Meanwhile, the oxygenator pressure drop and resistance were significantly higher in the group with duration of 6 days or longer (*p* < 0.001). In addition, both exhaust CO_2_ (*p* < 0.001) and oxygenator’s CO_2_ removal rate (*p* = 0.0189) were significantly lower in the longer duration group. Mechanical ventilation settings also were significantly different between the strata: the longer duration group had higher tidal volume (*p* = 0.027), respiratory rate (*p* < 0.001), minute ventilation (*p* < 0.001), and fraction of inspired oxygen (*p* = 0.031). Longitudinal trends in patient’s ventilation parameters are shown in Fig. 4.

**Table 2:** Exhaust carbon dioxide measurements and concomitantly recorded variable.

	All time points (N = 233)	Oxygenator duration shorter than 6 days (N = 116)	Oxygenator duration 6 days or longer (N = 117)	p-value*
Exhaust CO_2_ concentration (mmHg)	22.4 (18.1–33.0)	27.8 (20.1–37.2)	19.8 (16.9–26.2)	< 0.001
Blood flow rate (LPM)	4.40 (3.53–5.05)	4.28 (3.66–4.95)	4.47 (3.46–5.06)	0.617
Sweep gas flow rate (LPM)	5.0 (4.0–8.0)	5.0 (3.5–8.0)	6.0 (4.0–8.0)	0.169
CO_2_ Elimination Rate by the Oxygenator (mL/min)	175 (133–210)	180 (128–200)	171 (148–215)	0.0189
ECMO device V/Q ratio	1.26 (0.82–1.76)	1.22 (0.74–1.85)	1.29 (0.88–1.73)	0.324
Fraction of delivered oxygen	1.0 (1.0–1.0)	1.0 (1.0–1.0)	1.0 (1.0–1.0)	0.252
Oxygenator pressure drop (mmHg)	27 (20–31)	24 (19–29)	29 (24–35)	< 0.001
Oxygenator resistance (mmHg/L/min)	5.96 (5.19–6.82)	5.90 (4.42–6.37)	6.09 (5.45–7.32)	< 0.001
Patient temperature (celsius)	37.1 (36.8–37.3)	37.2 (36.9–37.4)	37.0 (36.8–37.3)	0.146
Tidal volume (mL)	318 (211–430)	281 (211–380)	360 (211–454)	0.027
Respiratory rate (breaths per minute)	24 (20–32)	22 (18–30)	28 (20–33)	< 0.001
Minute ventilation (LPM)	8.14 (4.83–11.56)	6.45 (4.28–10.51)	9.00 (5.80–12.60)	< 0.001
Fraction of inspired oxygen	0.6 (0.6–0.7)	0.6 (0.5–0.65)	0.6 (0.6–0.7)	0.031
Positive end-expiratory pressure (cmH2O)	10 (8–10)	10 (8–12)	10 (8–10)	0.051
Arterial blood gas results				
pH	7.38 (7.34–7.41)	7.39 (7.35–7.42)	7.36 (7.32–7.40)	0.001
PaCO_2_ (mmHg)	50 (45–61)	50 (44–59)	51 (45–62)	0.199
PaO_2_ (mmHg)	72 (64–87)	78 (65–96)	71 (64–80)	0.009
Serum hemoglobin (gm/dL)	8.0 (7.5–8.8)	8.6 (7.5–9.4)	7.8 (7.4–8.3)	< 0.001
Serum bicarbonate (mEq/L)	28.4 (25.0–36.0)	28.8 (26–35.8)	28.0 (25.0–37.0)	0.369
Serum lactate (mmol/L)	1.13 (0.81–1.49)	1.12 (0.75–1.48)	1.13 (0.98–1.49)	0.121
Oxygenator duration at exhaust CO_2_ measurement (days)	6.02 (2.81–10.34)	2.75 (1.06–4.0)	10.34 (7.88–17.88)	< 0.001
ECMO day at exhaust CO_2_ measurement (days)	12 (4–29)	4 (2–17)	21 (10–32)	< 0.001
Nautilus device measurements	125 (53.7)	56 (48.3)	69 (59.0)	0.103

CO_2_, Carbon dioxide; LPM, Liters per minute; PaCO_2_, Partial pressure of arterial carbon dioxide; PaO_2_, Partial pressure of arterial oxygen; ECMO, Extracorporeal membrane oxygenation

Data are presented as frequency with percentage or median with interquartile range

*Wilcoxon rank-sum

### Single variable correlations with exhaust CO_2_ concentration

**Fig. 5:**
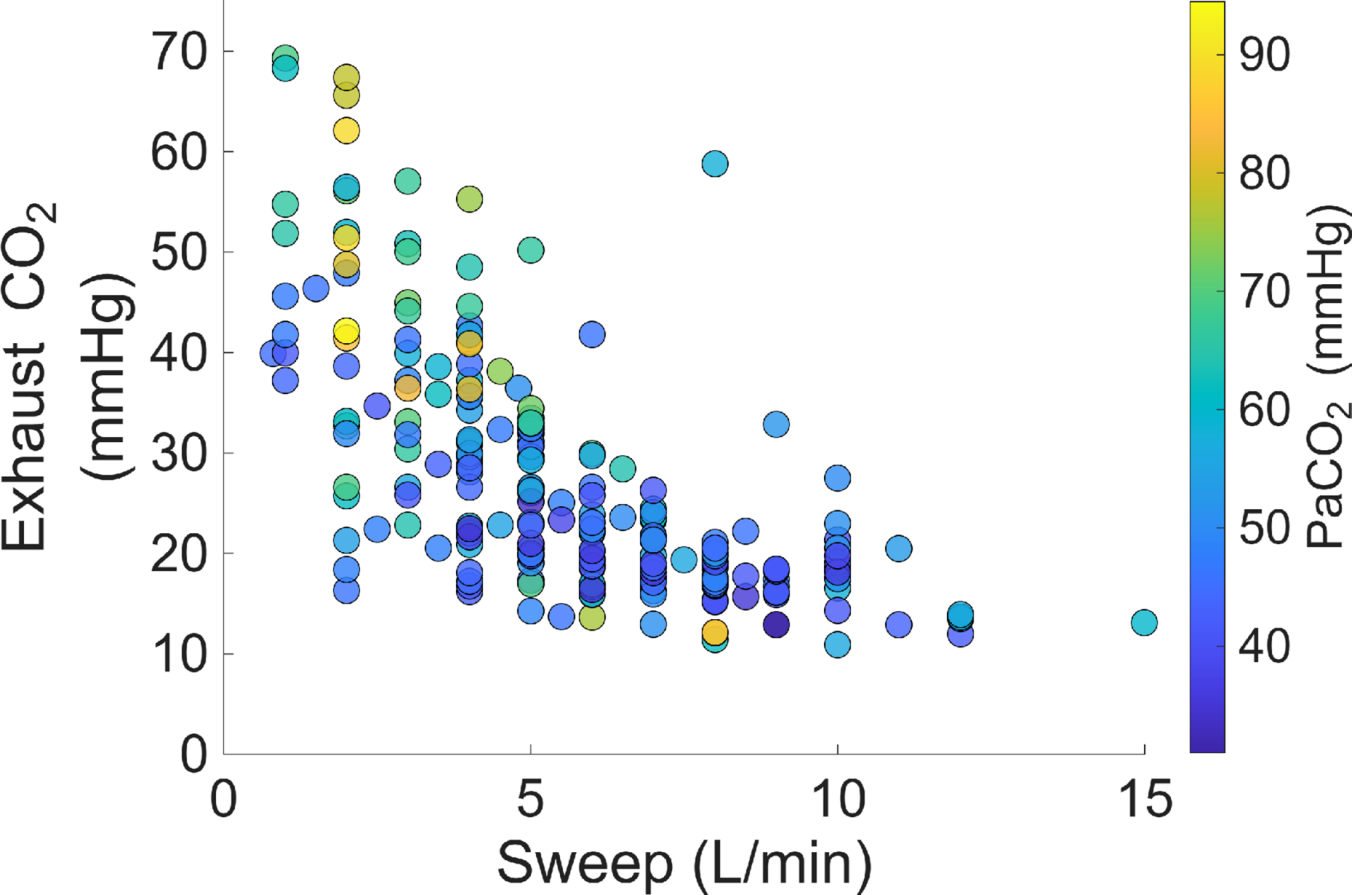
Unadjusted graphical presentation of the relationship between exhaust CO_2_ and sweep gas flow rate at the time of exhaust CO_2_ measurement. The concentration of the patient’s arterial CO_2_ at the time exhaust CO_2_ measurement is represented with color gradation.

Sweep gas flow rate demonstrated a strong, negative correlation with exhaust CO_2_ concentration (r = − 0.77; *p* < *0.001)*, as anticipated from prior work^16,17^. The unadjusted relationship between exhaust CO_2_ and sweep gas flow rate is presented in Fig. 5. Correlations of exhaust CO_2_ with duration of oxygenator use (r = − 0.44; *p* < *0.001)*, partial pressure of arterial CO_2_ (r = 0.38; *p* < *0.001),* and ECMO blood flow rate (r = 0.22; *p* = 0.02) were weak to moderate, as shown in Table 3. Non-ECMO respiratory support variables did not correlate with concentration of exhaust CO_2_, except for positive end-expiratory pressure (r = 0.33; *p* < 0.001), as shown in Table 3.

**Table 3:** Single variable correlations with exhaust CO_2_ concentration.

	All time points (N = 233)	Spearman’s coefficient	*p-value*
Exhaust CO_2_ concentration (mmHg)	22.4 (18.1–33.0)	1.00	
Blood flow rate (LPM)	4.40 (3.53–5.05)	0.22	0.022
Sweep gas flow rate (LPM)	5.0 (4.0–8.0)	− 0.77	< 0.001
ECMO device V/Q ratio	1.26 (0.82–1.76)	− 0.81	< 0.001
Fraction of delivered oxygen	1.0 (1.0–1.0)	− 0.08	0.399
Transmembrane pressure drop (mmHg)	27 (20–31)	0.06	0.552
Transmembrane pressure drop per Liter blood flow (mmHg/L)	5.96 (5.19–6.82)	− 0.15	0.122
Patient temperature (celsius)	37.1 (36.8–37.3)	0.09	0.362
Tidal volume (mL)	318 (211–430)	0.04	0.668
Respiratory rate (breaths per minute)	24 (20–32)	− 0.07	0.471
Minute ventilation (LPM)	8.14 (4.83–11.56)	− 0.07	0.468
Fraction of inspired oxygen	0.6 (0.6–0.7)	− 0.11	0.274
Positive end-expiratory pressure (cmH_2_O)	10 (8–10)	0.33	< 0.001
Carbon dioxide clearance (mL/min)	175.0 (133.5–210.0)	− 0.02	0.861
Arterial blood gas results			
pH	7.38 (7.34–7.41)	0.18	0.06
PaCO_2_ (mmHg)	50 (45–61)	0.38	< 0.001
PaO_2_ (mmHg)	72 (64–87)	− 0.02	0.864
Serum hemoglobin (gm/dL)#	8.0 (7.5–8.8)	0.31	0.001
Serum bicarbonate (mEq/L)	28.4 (25.0–36.0)	0.48	< 0.001
Serum lactate (mmol/L)	1.13 (0.81–1.49)	− 0.01	0.912
Oxygenator duration at exhaust CO_2_ measurement (days)	6.02 (2.81–10.34)	− 0.44	< 0.001
ECMO day at exhaust CO_2_ measurement (days)	12 (4–29)	− 0.31	0.001

CO2*,* Carbon dioxide; LPM*,* Liters per minute; PaCO2*,* Partial pressure of arterial carbon dioxide; PaO2*,* Partial pressure of arterial oxygen; ECMO*,* Extracorporeal membrane oxygenation.

### Multivariable regression

In a multivariable, mixed-effects linear regression model, exhaust CO_2_ decreased as sweep gas flow increased (Table 4). The magnitude of the effect of sweep gas flow on exhaust CO_2_ concentration was greater when sweep gas flow was less than 4 LPM (adjusted β, − 5.556; 95% CI, − 6.886 to − 4.226; *p* < 0.001) relative to 4–8 LPM (adjusted β, − 1.642; 95% CI, − 2.394 to − 0.897; *p* < 0.001) and greater than 8 LPM (adjusted β, − 1.391; 95% CI, − 2.599 to − 0.175; *p* = 0.003). Exhaust CO_2_ concentration increased as ECMO blood flow increased (adjusted β, 2.341; 95% CI, 1.041 to 3.633; *p* < 0.001) as shown in Table 4.

**Table 4:** Multivariable mixed-effects linear regression examining factors that affect exhaust carbon dioxide concentration.

Variable	Estimate (β)	95% CI	*p-value*
Sweep gas flow rate (L/min)			
≤ 4	− 5.556	− 6.886, − 4.226	< 0.001
> 4–8	− 1.642	− 2.394, − 0.897	< 0.001
> 8	− 1.391	− 2.599, − 0.175	0.003
ECMO blood flow rate (L/min)	2.341	1.041, 3.633	< 0.001
Duration of oxygenator use at the time of the observation (days)	− 0.258	− 0.426, − 0.099	0.002
Arterial CO_2_ (mmHg)	0.175	0.061, 0.281	0.002
CardioHelp oxygenator (reference: Nautilus oxygenator)	1.756	− 2.751, 6.255	0.45

ECMO*,* Extracorporeal membrane oxygenation; CO_2_*,* Carbon dioxide.

Additionally, holding other variables constant, both duration of oxygenator use (adjusted β − 0.258; 95% CI, − 0.426 to − 0.099; *p* = 0.002) and partial pressure of arterial CO_2_ (adjusted β, 0.175; 95% CI, 0.061 to 0.281; *p* = 0.002) affected measured exhaust CO_2_ concentration, though the size of these effects were smaller than either the sweep gas flow rate or ECMO blood flow rate. Oxygenator type was not associated with differences in exhaust CO_2_ concentration, when controlled for other variables (adjusted β, 1.756; 95% CI, − 2.751 to 6.255; *p* = 0.45).

## Discussion

To the authors’ knowledge, this is the first study reporting longitudinal membrane oxygenator exhaust CO_2_ measurements and the relationships between CO_2_ removal and other important clinical variables in human subjects receiving VV-ECMO across days to weeks of oxygenator use. These data comprise exhaust CO_2_ measurements over a broad range of sweep gas flow, oxygenator duration, and partial pressure of arterial CO_2_, which strengthened the ability to test the effect of these variables on exhaust CO_2_ concentration across values observed during VV-ECMO.

Correlations between exhaust CO_2_, sweep gas flow rate, ECMO blood flow rate, and membrane oxygenator duration agreed with physiologic principles and prior mechanistic work^8,11,16,17^. Exhaust CO_2_ significantly decreased with increasing sweep gas flow rate, which is primarily explained by the shorter residence time with increasing sweep and hence reduced accumulation of CO_2_ along the gas pathway^18,19^. The findings also agreed with prior work that demonstrated sweep gas flow rate as the most important factor determining CO_2_ elimination for a given oxygenator type^8,20,21^. Increasingly, clinicians recognize that ECMO blood flow rate plays a role in CO_2_ elimination, particularly at higher rates of sweep gas flow^11,17,22^. Carbon dioxide removal is generally considered to be a sweep-limited process, so increasing sweep is the direct straightforward way of removing more CO_2_. Only when the sweep gas flow reaches maximum rate does this process start to be limited by the blood flow. The multivariate analysis also demonstrated that there was a measurable effect of ECMO blood flow rate on CO_2_ removal. For patients that are refractory to maximum sweep gas flow, blood flow may be increased to achieve additional CO_2_ clearance. However, CO_2_ clearance is not regularly measured or documented during clinical routine ECMO, so additional clinical data are needed to further support this claim. Additionally, multivariate analysis also showed that CO_2_ removal rate decreased with longer duration of oxygenator use and increasing minute ventilation rate. The authors speculate that this finding may be explained by two potential causes: oxygenator may be losing efficiency of CO_2_ removal due to thrombus burden, and/or mechanical ventilator and membrane oxygenator are competing for available CO_2_. Oxygenator pressure drop and resistance were significantly higher in the longer duration group than the shorter duration (Table 2), which aligns with our theory about the clot burden reducing oxygenator efficiency. Further studying and differentiating these potential causes in future studies will be necessary before reliably using exhaust CO_2_ and CO_2_ elimination rate to inform clinical management of VV-ECMO.

Prior publications on the topic of exhaust CO_2_ monitoring studied it in the context of cardiopulmonary bypass to estimate the partial pressure of arterial CO_2_^12,23^. There were also preclinical large animal ECMO models that used exhaust CO_2_ readings inform and autoregulate sweep gas flow titration^13^. These applications rely on identifying and quantifying the independent effect of partial pressure of CO_2_ in blood on the measured concentration of exhaust CO_2_. To this end, this study provides additional clinical data to further elucidate this relationship between exhaust and blood CO_2_. Unadjusted and adjusted analysis here indeed showed a significant relationship between PaCO_2_ and exhaust CO_2_ (Tables 3 and 4). Previous work has also examined the potential for exhaust CO_2_ monitoring to identify changes in membrane function earlier, either due to slow increases in oxygenator dead space or acute oxygenator thrombosis^10^. Our findings potentially add to this prior work by providing exhaust CO_2_ data with clinical VV-ECMO data correlates. The opportunity remains to develop a more thorough understanding of these relationships, so that clinicians can better isolate the etiology of an observed change in exhaust CO_2_ amidst the dynamic clinical circumstances and management of ECMO patients.

There are multiple limitations to this study. First, there are a few limitations related to data acquisition methods. Sweep gas flow rate was recorded from a visual readout of the sweep gas flow rotameters, which is less precise than digital measurements. Additionally, the exhaust CO_2_ was measured by attaching an air pump to divert exhaust gas, and this reading could get diluted or contaminated by the ambient air. More advanced methods of sweep gas flow and exhaust CO_2_ measurement would improve the reliability of these readouts^11,24^. We also did not collect information that we know to affect extracorporeal CO_2_ removal. We did not measure pre-oxygenator blood partial pressure of CO_2_ or O_2_ at the time of exhaust CO_2_ measurements, as we do not routinely maintain pre-oxygenator access for blood sampling at our institution. Without this information, it is difficult to relate CO_2_ removal data to CO_2_ concentration gradient. Currently, the institution uses noninvasive perfusion monitoring technology that can measure oxyhemoglobin saturation at device inlet and outlet, so future studies can better correlate exhaust CO_2_ measurements with real-time values of blood oxygenation. We also did not record end-tidal CO_2_ readings from the mechanical ventilators for this study. Future studies will study and compare the rates of CO_2_ removal happening in the native lungs versus the extracorporeal artificial lungs. We did not measure or correct for the content of CO_2_ in sweep gas flow entering the oxygenator, since we assumed this to be zero. Most of the presented data were obtained while the fraction of delivered oxygen to device was 100%, but we acknowledge that there may be a nonzero amount of CO_2_ present, especially in room air blended with oxygen in the sweep. Finally, the reported values are only steady-state measurements, so there is future opportunity to investigate the clinical utility of analyzing transient changes in exhaust CO_2_.

## Conclusion

Our findings suggest there may be a role for non-invasive monitoring of exhaust CO_2_ during VV-ECMO and other forms of ECLS. Significant relationships between sweep gas, blood flow, duration of use, minute ventilation, and CO_2_ removal rate were observed. These data lay a framework for additional investigation to clarify the multiple factors that affect measurements of exhaust CO_2_ to derive clinical utility of this cost-efficient, non-invasive measurement. Accurate exhaust CO_2_ and sweep gas flow sensors, combined with strict protocols for consistently obtaining exhaust CO_2_ measurements, are necessary to maximize precision of measurements if exhaust CO_2_ is to enhance clinical decision-making. As several variables affect exhaust CO_2_ concentration and thus CO_2_ clearance, exhaust CO_2_ measurements alone are unlikely to inform clinical diagnosis of the oxygenator efficiency or to guide sweep gas flow titration. Thus, monitoring trends over time could offer additional utility, but further study is needed to generate the data necessary to interpret longitudinal trends. Additionally, there may be potential for decision support algorithms and software that rapidly integrate multiple variables to aid clinical interpretation of isolated measurements or continuous trends of exhaust CO_2_. Robust data from larger patient cohorts are necessary to develop such systems.

## Methods

### Study design

A single-center prospective cohort study was conducted among adult patients receiving VV-ECMO at Vanderbilt University Medical Center between April 1, 2021, and June 15, 2021. This study was approved by the Vanderbilt University Medical Center’s Human Research Protections Program and its Institutional Review Board (IRB, Protocol Number 210226). Given the impact of ECMO on the patient’s cognitive function, the study team obtained informed written consent from an appropriate surrogate for study participation. All methods were performed in accordance with the relevant federal and institutional guidelines and regulations.

### Location and participants

All adults (18 years of age or older) receiving VV-ECMO at Vanderbilt University Medical center during the study period were eligible. Exclusion criteria were patients with more than one membrane oxygenator incorporated into their ECMO circuit and presence of an arterial reinfusion cannula. Eligible patients were enrolled immediately upon completion of informed consent at ECMO initiation. Patients whose ECMO support was initiated at a hospital outside of Vanderbilt were enrolled upon admission to an ICU at our institution.

### ECMO circuit equipment and management

Initiation of VV-ECMO reflected Extracorporeal Membrane Oxygenation for Severe Acute Respiratory Distress (EOLIA) trial inclusion criteria^25^. All patients were supported with either a Cardiohelp HLS set advanced 7.0 (Getinge, Gothenburg, Sweden) circuit and oxygenator or a Nautilus oxygenator (Medtronic, Dublin, Ireland) integrated within a CentriMag (Abbott Laboratories, Chicago, IL) blood pump and circuit. Both the Cardiohelp HLS set advanced 7.0 and Nautilus oxygenators employed a polymethylpentene membrane with equivalent gas exchange surface areas of 1.8 square meters. Clinical decisions, including indications and timing of circuit exchanges, timing of ECMO decannulation, ECMO support settings, and non-ECMO respiratory support settings were determined by the clinical teams and not influenced by study interventions or measured data.

### Study interventions and data collection

The study investigators intermittently measured exhaust CO_2_ concentration from the membrane oxygenators of study participants. Time points for exhaust CO_2_ measurements were selected to reflect steady-state conditions (i.e. measurements were not obtained immediately surrounding changes to ECMO or non-ECMO respiratory support settings). All exhaust CO_2_ measurements were obtained using a K33 ICB 10% CO_2_ gas analyzer kit (CO2METER.com, Ormond Beach, FL), which included a miniature air pump for gas sampling and CO_2_ gas analyzer rated up to 10% concentration. The device used for exhaust CO_2_ measurements is not approved by the Food and Drug Administration for clinical use. As such, it was clarified during the IRB approval process that measurements obtained during the study were not shared with the clinical ECMO team or used to influence clinical decision-making. The CO_2_ meter was attached to the oxygenators of study participants using ¼-inch tubing in a side-stream fashion from the exhaust port of the oxygenator, with additional tubing in-line with exhaust port to prevent room air contamination of measurements^26^, as shown in Fig. 1. Prior to measurements, excess water in the gas chamber was purged, by briefly increasing sweep gas flow to maximum for 1 min, bringing back down to original value, and waiting until the real-time exhaust CO_2_ reading reaches a steady state. Real-time CO_2_ concentration readings from the gas analyzer were displayed using Gas Lab software (CO2METER.com, Ormond Beach, FL), and steady-state time point measures of CO_2_ concentration were recorded in Research Electronic Data Capture (REDCap).

In conjunction with exhaust CO_2_ concentration measurements, the following variables were also simultaneously collected: ECMO day, oxygenator duration, ECMO settings (blood flow rate, sweep gas flow rate, fraction of delivered oxygen, and transmembrane pressure), mechanical ventilator settings (tidal volume, respiratory rate, fraction of inspired oxygen, positive end-expiratory pressure), arterial blood gas values, serum bicarbonate, hemoglobin concentration, and patient temperature. Demographics, baseline characteristics, and outcomes were extracted from the electronic medical record. Data collections were conducted through REDCap.

### Statistical analysis

Baseline characteristics and timepoint variables were first examined with descriptive statistics. Continuous variables were expressed as median and interquartile range and categorical variables were expressed as frequency and percentage. Exhaust CO_2_ measurements and associated variables collected at the time of data collection were stratified by less than or greater to 6 days of oxygenator use and by oxygenator type. The Wilcoxon rank-sum test was used to examine between-group differences in continuous variables. Linearity between exhaust CO_2_ and candidate effect variables was examined by scatter plot and by Shapiro–Wilk and Cameron-Trivedi decomposition tests for normality and homogeneity of residuals of simple linear regression plots. Correlation between collected variables and exhaust CO_2_ concentration were examined using Spearman’s correlation coefficient given predominance of non-linear or ordinal data. Then, multivariable mixed-effects linear regression was performed, which specified patient and oxygenator as random-effects, and exhaust CO_2_ as the outcome. Variables that were considered clinically meaningful and correlated with exhaust CO_2_ concentration in the single variable correlation analysis were selected as fixed-effect variables: sweep gas flow rate, blood flow rate, partial pressure of arterial CO_2_, and duration of oxygenator use at the time of exhaust CO_2_ measurement. We used linear splines with 2 knots to account for the nonlinear relationship between sweep gas flow and exhaust CO_2_, which was expected based on prior work^11,16^ and confirmed by previously described assessments for linearity. Analyses were performed using STATA 14.2 (College Station, TX).

## Data Availability

Data are available upon request.

## Abbreviations

ARDS: Acute respiratory distress syndrome
VV-ECMO: Venovenous extracorporeal membrane oxygenation
COVID-19: Coronavirus disease 2019
CO_2_: Carbon dioxide
ICU: Intensive care unit
IRB: Institutional review board
EOLIA: Extracorporeal membrane oxygenation for severe acute respiratory distress syndrome
